# Co‐occurrence of neurofibromatosis type 1 and optic nerve gliomas with autosomal dominant polycystic kidney disease type 2

**DOI:** 10.1002/mgg3.1321

**Published:** 2020-06-13

**Authors:** Ramón Peces, Rocío Mena, Yolanda Martín, Concepción Hernández, Carlos Peces, Dolores Tellería, Emilio Cuesta, Rafael Selgas, Pablo Lapunzina, Julián Nevado

**Affiliations:** ^1^ Servicio de Nefrología Hospital Universitario La Paz IdiPAZ Universidad Autónoma Madrid Spain; ^2^ Instituto de Genética Médica y Molecular (INGEMM)‐IdiPAZ Hospital Universitario La Paz Universidad Autónoma Madrid Spain; ^3^ CIBERER, Centro de Investigación Biomédica en Red de Enfermedades Raras ISCIII Madrid Spain; ^4^ Servicio de Genética Hospital Universitario Ramón y Cajal Centro de Investigación Biomédica en Red de Enfermedades Raras (CIBERER) Madrid Spain; ^5^ Area de Tecnologías de la Información SESCAM Toledo Spain; ^6^ Servicio de Radiología Hospital Universitario La Paz IdiPAZ Universidad Autónoma Madrid Spain

**Keywords:** autosomal dominant polycystic kidney disease (ADPKD), neurofibromatosis type 1 (NF1), next‐generation sequencing (NGS), NF1 mutation, optic pathway gliomas, PKD2

## Abstract

**Background:**

Autosomal dominant polycystic kidney disease (ADPKD) and neurofibromatosis type 1 (NF1) are both autosomal dominant disorders with a high rate of novel mutations. However, the two disorders have distinct and well‐delineated genetic, biochemical, and clinical findings. Only a few cases of coexistence of ADPKD and NF1 in a single individual have been reported, but the possible implications of this association are unknown.

**Methods:**

We report an ADPKD male belonging to a family of several affected members in three generations associated with NF1 and optic pathway gliomas. The clinical diagnosis of ADPKD and NF1 was performed by several image techniques.

**Results:**

Linkage analysis of ADPKD family was consistent to the *PKD2* locus by a nonsense mutation, yielding a truncated polycystin‐2 by means of next‐generation sequencing. The diagnosis of NF1 was confirmed by mutational analysis of this gene showing a 4‐bp deletion, resulting in a truncated neurofibromin, as well. The impact of this association was investigated by analyzing putative genetic interactions and by comparing the evolution of renal size and function in the proband with his older brother with ADPKD without NF1 and with ADPKD cohorts.

**Conclusion:**

Despite the presence of both conditions there was not additive effect of *NF1* and *PKD2* in terms of the severity of tumor development and/or ADPKD progression.

## Graphical abstract

We report an Autosomal dominant polycystic kidney disease (ADPKD) male belonging to a family of several affected members in three generations associated with neurofibromatosis type 1 (NF1) ) and optic pathway gliomas. Conclusión: this is the first reported case of concurrence of a truncated NF1 mutation with optic pathway gliomas and a nonsense PKD2 mutation. Despite the presence of both conditions there was not additive effect of NF1 and PKD2 in terms of the severity of tumour development and/or ADPKD progression.

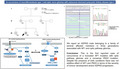

## 1 INTRODUCTION

Autosomal dominant polycystic kidney disease (ADPKD) and neurofibromatosis type 1 (NF1) (MIM#: 162200) are both inherited as autosomal dominant disorders (Bergmann et al., 2018; Stewart, Korf, Nathanson, Stevenson, & Yohay, 2018; Torres, Harris, & Pirson, 2007; Ward & Gutmann, 2005). ADPKD is the most common hereditary kidney disease, resulting from mutations in *PKD1* (MIM#: 601313) and *PKD2* (MIM#: 173910) genes, located in chromosomes 16p13.3 (in approximately 85% of cases) and 4q22.1 (in approximately 15% of cases), respectively. A rarer and newly discovered third locus including *GANAB* (MIM#: 104160) gene (at 11q12.3); is also associated with autosomal dominant polycystic liver disease in <1% of cases (Besse et al., 2018). In ADPKD, the growth of renal cysts produces a progressive increase in renal volume and destruction of the parenchyma, resulting in renal failure in the majority of affected individuals. However, disease progression of ADPKD is highly variable, in part because of a strong gene locus effect (Audrézet et al., 2016; Magistroni et al., 2003; Rossetti et al., 2002; Stewart et al., 2018), or by a possible modifier effect as it is suggested by a significant intra‐familial renal disease variability (Harris & Rossetti, 2010). Polycystin‐1 and polycystin‐2, the proteins encode by *PKD1* and *PKD2* genes, respectively, are involved in many functions of cell proliferation through the cellular GTPase systems (Delmas et al., 2002; Gogusev et al., 2003; Kim et al., 1999). NF1 is also relatively common and is caused by a mutation in the *NF1* gene (MIM#: 613113), which is present on chromosome 17q11.2 (Abramowicz & Gos, 2014; Hirbe & Gutmann, 2014; Ward & Gutmann, 2005). This gene codes for a protein known as neurofibromin, which is involved in inhibiting the cell proliferation (Abramowicz & Gos, 2014; Hirbe & Gutmann, 2014; Ward & Gutmann, 2005). Both diseases are known to co‐exist with a number of other hereditary diseases, such as tuberous sclerosis, Von Hippel‐Lindau, Marfan disease, Noonan syndrome, hereditary breast cancer, pheochromocytoma, or multiple endocrine neoplasia type 2 (Alaraj et al.., 2007; Campos et al., 2013; Chatha et al., 2001; Erbay, Oljeski, & Bhadelia, 2004; Ercolino et al., 2014; Hateboer, Buchalter, Davies, Lazarou, & Ravine, 2000; Martignoni et al., 2002; Oktenli, Gul, & Deveci, 2004; Prada et al., 2011; Sampson et al., 1997; Syro et al., 2012; Thiel et al., 2009; Wheeler & Sadeghi‐Nejad, 2005). However, co‐occurrence of these two diseases (ADPKD and NF1) in a single individual is extremely rare. To the best of our knowledge, only a few cases have been reported in the literature (Table 1; Chen, Chen, Hou, Lee, & Huang, 2002; Flego et al., 2003; Niemczyk et al., 2010; Siegelman, Zavod, & Hecht, 1971; Varma, Talwar, Kushik, & Sharma, 1982). In only one of these cases, there was inheritance of one condition, ADPKD, and sporadic case and de novo mutation of the other, at NF1, was assumed (Chen et al., 2002). However, an association of NF1 with optic pathway gliomas and ADPKD confirmed by molecular genetic analysis of the two genes has not been previously described.

**TABLE 1:** List of published series of patients with concurrence of ADPKD and NF1

Author	Year	*N*	Family history ADPKD	Family history NF1	Age, years	Sex
Siegelman	1971	1	No	Yes	54	M
Varma	1982	1	No	No	40	F
Chen	2002	3	Yes	Yes	47, 18, 17	F, M, M
Flego	2003	1	Yes	Yes	50	M
Current report	2019	1	Yes	No	30	M

Abbreviations: ADPKD, Autosomal dominant polycystic kidney disease; *N*, number of patients; NF1, neurofibromatosis type 1.

Herein, we report a case of an ADPKD family, in which co‐occurrence of pathogenic *PKD2* and *NF1* mutations in a single individual has been revealed based on clinical and genetic studies. We characterize the genetic background in the proband and his family members and discuss the putative genotype‐phenotype correlations. Due to the influence that may have each disorder on the other, we also analyse possible implications of the presence of *PKD2* and *NF1* in the same individual and the possibility that this might have resulted in putative genetic interactions and/or additive or synergistic effects through biochemical pathways. Finally, we compared the clinical evolution of this rare case with his older brother with isolated ADPKD and with others control ADPKD populations followed up by our center. To our knowledge, this is the first reported case of NF1 associated with ADPKD subjected to clinical and extensive molecular studies.

## 2 MATERIALS AND METHODS

### 2.1 Case presentation

**FIGURE 1:**
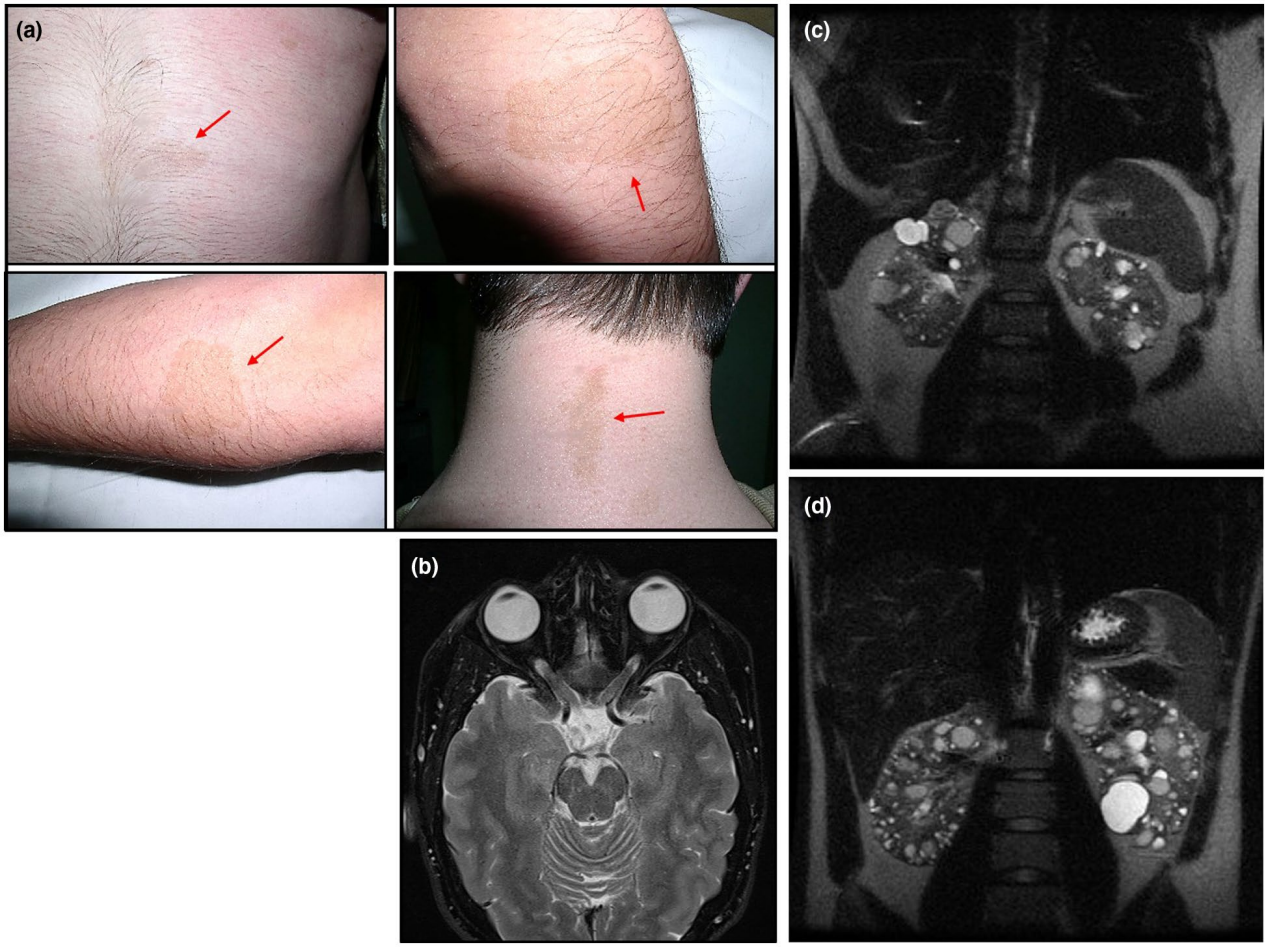
Clinical features for concurrence of NF1 and ADPKD in the proband. (a) Presence of café‐au‐lait spots over neck, trunk, and extremities. (b) Axial MRI at the age of 17 years showing bilateral thickening of the optic nerve and chiasm. There were focal areas of increased signal intensity on T2 in the bilateral globus pallidus and hippocampus. (c) Coronal T2‐weighted MRI scan of the abdomen in the proband at the age of 28 years showing a TKV of 587 ml (htTKV of 360 ml/m). (d) Coronal T2‐weighted MRI scan of the abdomen in his brother at the age of 30 years showing a TKV of 791 ml (htTKV of 437 ml/m). ADPKD, Autosomal dominant polycystic kidney disease; htTKV, height adjusted total kidney volume; MRI, magnetic resonance imaging; NF1, neurofibromatosis type 1; TKV, total kidney volume

A 17‐year‐old male with maternal familial history of ADPKD visited our outpatient clinic for renal evaluation in October 2005. He was the second child of unrelated parents. His past medical history was significant for NF1, diagnosed (using NIH consensus criteria) (NIH, 1988) in infancy by the presence of several café‐au‐lait spots in different part of his body, as well as axillary and inguinal frecklings. The patient also had optic pathway gliomas since age 5, which was documented by magnetic resonance imaging (MRI) serial studies. Visual field testing and visually evoked potentials were also confirmatory. In addition, an ophthalmologic examination at age of 16 years old revealed a decrease of visual field. Physical examination at the time of renal evaluation revealed a normal blood pressure and the presence of multiple café‐au‐lait spots over trunk, neck, and extremities (Figure 1a). Axillary and inguinal frecklings were also found. He had a serum creatinine of 0.75 mg/dl and estimated glomerular filtration rate (eGFR) of 111 ml/min/1.73 m^2^. An ultrasonography (US), a computed tomography (CT), and a MRI of the abdomen showed both kidneys with several cysts of variable size. A cranial MRI demonstrated optic pathway gliomas involving the optic nerves and chiasm (Figure 1b). There were focal areas of increased signal intensity on T2 in the bilateral globus pallidus, hippocampus, amygdala, cerebellar hemispheres, and periventricular white matter. It was considered that he did not require specific treatment. Because the familial history of cerebral hemorrhage, MRI angiography screening for intracranial aneurysms was performed at age of 17 and 20 years, respectively, yielding both negative results. At age of 22 years old he had a serum cystatin C 0.68 mg/dl and estimated glomerular filtration rate (eGFR_CysC_) of 141 ml/min/1.73 m^2^. Total kidney volume (TKV) and height adjusted total kidney volume (htTKV) determined by MRI were measured by manual segmentation (Bae, Commean, & Lee, 2000). The patient had a TKV of 521 ml and an htTKV of 320 ml/m, and 6 years later (at age of 28) the TKV was 587 ml and htTKV 360 ml/m (Figure 1c) (an annual increase of 2.1%). Currently, he has a normal blood pressure, a serum cystatin C of 0.69 mg/dl and eGFR_CysC_ of 111 ml/min/1.73 m^2^ (Table 2). There was no clinical evidence of the presence of cutaneous neurofibromas, plexiform neurofibromas, pheochromocytomas or other tumor types. In this way, he had negative results in serial abdominal and pelvic US, CT, and MRI.

**TABLE 2:** Clinical features of the proband, his older brother and three different ADPKD cohorts

Parameter	Proband	Brother	PKD2 patients (*N* = 12, mean ± *SD*)	ADPKD patients without HT (*N* = 23, mean ± *SD*)	ADPKD patients ≤ 40 years old (*N* = 58, mean ± *SD*)
Age, years	28	30	38 ± 6.4	35 ± 5.6	33.1 ± 6.3
TKV, ml	587	791	727 ± 359	662 ± 223	1,301 ± 966
HtTKV, ml/m	360	437	428 ± 205	382 ± 124	752 ± 563
Annual TKV, %	2.1	4.2	5.5 ± 2.3	5.6 ± 4.4	7.3 ± 5.3
eGFR_CysC_, ml/min/1.73 m^2^	111	118	112 ± 13	137 ± 19	119 ± 30

Abbreviations: ADPKD, Autosomal dominant polycystic kidney disease; HT, hypertension; HtTKV, height adjusted total kidney volume; TKV, total kidney volume.

### 2.2 Family history

There was no family history of NF1, but the family history was positive for ADPKD. All available members of the family were screened at least once by abdominal US, CT, or MRI and the diagnosis of ADPKD was confirmed according to recognized criteria (Pei et al., 2009). Reviewing the family history, maternal grandmother, mother, maternal aunt, and older brother of the proband were affected by ADPKD. His maternal grandmother presented end stage renal disease (ESRD) from ADPKD at age of 70 years and deceased from a cerebral hemorrhage at the age of 71 years. His maternal aunt was found to have also ADPKD at 37 years of age and presented ESRD at age of 60. His mother was found to have ADPKD at 35 years of age and presented ESRD at age of 49. His older brother was found to have bilaterally enlarged kidneys with several cysts (6–8) of variable size at age of 14 years. At age of 24 he has a serum cystatin C of 0.65 mg/dl and eGFR_CysC_ of 149 ml/min/1.73 m^2^; TKV was 634 ml and HtTKV 350 ml/m, and 6 years later (at age of 30) the TKV was 791 ml and HtTKV 437 ml/m (Figure 1d) (an annual increase of 4.2%). Currently, he has a normal blood pressure, a serum cystatin C of 0.79 mg/dl and eGFR_CysC_ of 118 ml/min/1.73 m^2^ (Table 2). His younger brother currently with 28 years of age showed no evidence of ADPKD so far. MRI angiography screening for intracranial aneurysms of all family members with ADPKD was negative.

### 2.3 Follow‐up study

To document the evolution of ADPKD, MRI, and eGFR serial studies in both brothers and three control ADPKD cohorts (among the patients with ADPKD followed up by our center) were performed. To this end, TKV and htTKV were also determined in these ADPKD cohorts. The eGFR was determined using the serum creatinine and the serum cystatin C values. Cystatin C level was used to calculate eGFR using the formula: eGFR_CysC_ = 77.24 × (Cys^−1.2623^). Mayo clinic imaging classification and the prediction of future eGFR based on the classification were determined in both brothers (Irazabal et al., 2015). In addition, we compared the clinical evolution of the proband with his older brother with ADPKD only and with three control ADPKD defined cohorts: (PKD2 patients, *N* = 12), (ADPKD without hypertension, *N* = 23) and (ADPKD ≤ 40 years, *N* = 58) (Table 2).

### 2.4 Molecular genetics

#### 2.4.1 ADPKD by microsatellite analysis

We initially ascertained the complete pedigree of the family over three generations and clinically assessed and genotyped all available members with polymorphic markers at *PKD1* and *PKD2* locus. Haplotype analysis by microsatellite markers KG8 (intragenic) and OT2 (D16S521) for *PKD1* and D4S1534, JV106, D4S1563, and D4S423 for *PKD2* were done (Coto et al., 1995).

#### 2.4.2 ADPKD by multiplex ligation‐dependent probe amplification analysis

Autosomal dominant polycystic kidney disease gross deletion analysis were made by means of multiplex ligation‐dependent probe amplification (MLPA) using the SALSA MLPA KIT P351/P352 for *PKD1/PKD2* (MRC‐Holland), following manufacturer´s instructions. Data analysis were done with Coffalyser v6 MLPA Analysis Software (MRC‐Holland).

#### 2.4.3 ADPKD massive parallel sequencing analysis

The use of massive parallel sequencing technologies for in‐depth analysis of large numbers of genes simultaneously is a time‐efficient and a cost‐effective manner, and the advantages of its use in diagnostic screening of human diseases, including renal diseases, have recently been demonstrated (Harris, 2018). We used a custom‐targeted next‐generation sequencing (NGS) gene panel (NEFROseq v1.1) for the study of the full spectrum of genetic nephropathies (which includes 250 genes implicated in etiology of kidney diseases). The sequence was captured using SeqCap EZ technology (Roche Nimblegen) and subsequently sequenced on a NextSeq500 (Illumina). Bioinformatics analysis was performed in the house Bioinformatics’ core using publicly available software tools (trimmomatic‐0.32; Bowtie2 v2.1.0; Picard‐tools v1.27; Samtools v0.1.19‐44428cd; Bedtools v2.26.0; Genome Analysis TK v3.3‐0 y SnpE 4.1l; ClinVar date 20140703 dbscSNV1.1 dbNSFP version 3.0 dbSNP v138), and simultaneously with variant Caller V2.1 tool (Illumina). In silico pathogenicity prediction was analyzed using Alamut 2.7 (interactive Biosoftware) and others such as SIFT ensembl 66; Polyphen‐2 v2.2.2; Mutation Assessor, release 2; FATHMM, v2.3; Gerp2; PhyloP; CADD, v1.3. Population frequencies of the detected variants were assessed using the Exome Aggregation Consortium (ExAC; Exac r0.3) data (http://exac.broadinstitute.org); 1000 genome project; Spanish Exon Variant Project; NHLBI exome sequencing project: ESP6500_EA_AF; Candidate variants in the proband were subsequently confirmed by Sanger sequencing, as was family testing.

#### 2.4.4 Sanger sequencing

*PKD2* mutation screening for coding sequences and intron/exon boundaries for exon 8 (*PKD2*; NM_000297.3) was performed by direct sequencing. PCR conditions and Primers (designed with the help of Primer3 plus v04.0 Software) were available upon request. PCR products were sequenced using BrightDye Terminator cycle kit (Nimagen) and run on a ABI3730XL Sequencer (Thermo Fisher).

#### 2.4.5 Complete NF1 mutational analysis

Comprehensive *NF1* gene mutation screen involved MLPA analysis and mRNA‐based mutation detection by RT‐PCR and denaturing high‐performance liquid chromatography (DHPLC) (Valero et al., 2011; Valero, Velasco, Moreno, & Hernández‐Chico, 1994). Genomic DNA was purified from peripheral blood leucocytes by standard procedure and RNA was extracted using QIAamp^®^ RNA Blood kit (QIAGEN). Screening for *NF1* single and multi‐exon deletions was performed using the SALSA MLPA KIT P081/P082 *NF1* (MRC‐Holland), as instructed by the manufacturer. Data analysis were done with Coffalyser v6 MLPA Analysis Software (MRC‐Holland). RNA was reverse transcribed using First Strand cDNA Synthesis Kit for RT‐PCR (AMV) and random hexamers (Roche). The entire coding region of *NF*1 gene was amplified in 23 overlapping fragments and DHPLC was performed on a WAVE DNA fragment analysis system using a DNAsep column (Transgenomic). Variant profiles were characterized by sequencing the product of a second PCR amplification using an ABI PRISM 3100 Genetic Analyser (Applied Biosystems). Mutation identified was confirmed in genomic DNA by direct sequencing. Mutation and its putative effect at the protein level have been named according to the Human Genome Variation Society guidelines (den Dunnen & Antonarakis, 2003). Mutation numbering is based on the *NF1* mRNA sequence from Genbank (NM_000267.2), with the A of the translation start codon considered as nucleotide number 1. Exons are not named consecutively but according to the accepted nomenclature used by researchers in the field.

## 3 RESULTS

### 3.1 ADPKD family

**FIGURE 2:**
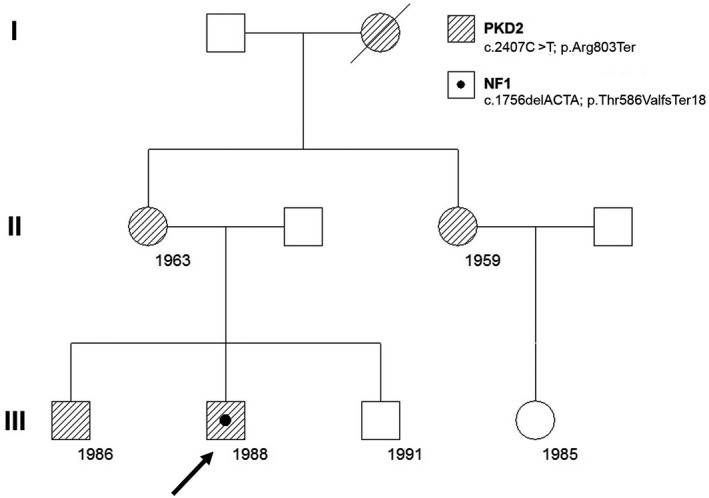
Pedigree of the family with PKD2 and NF1. The number is the year of birth. The arrow shows the proband. NF1, neurofibromatosis type 1

**FIGURE 3:**
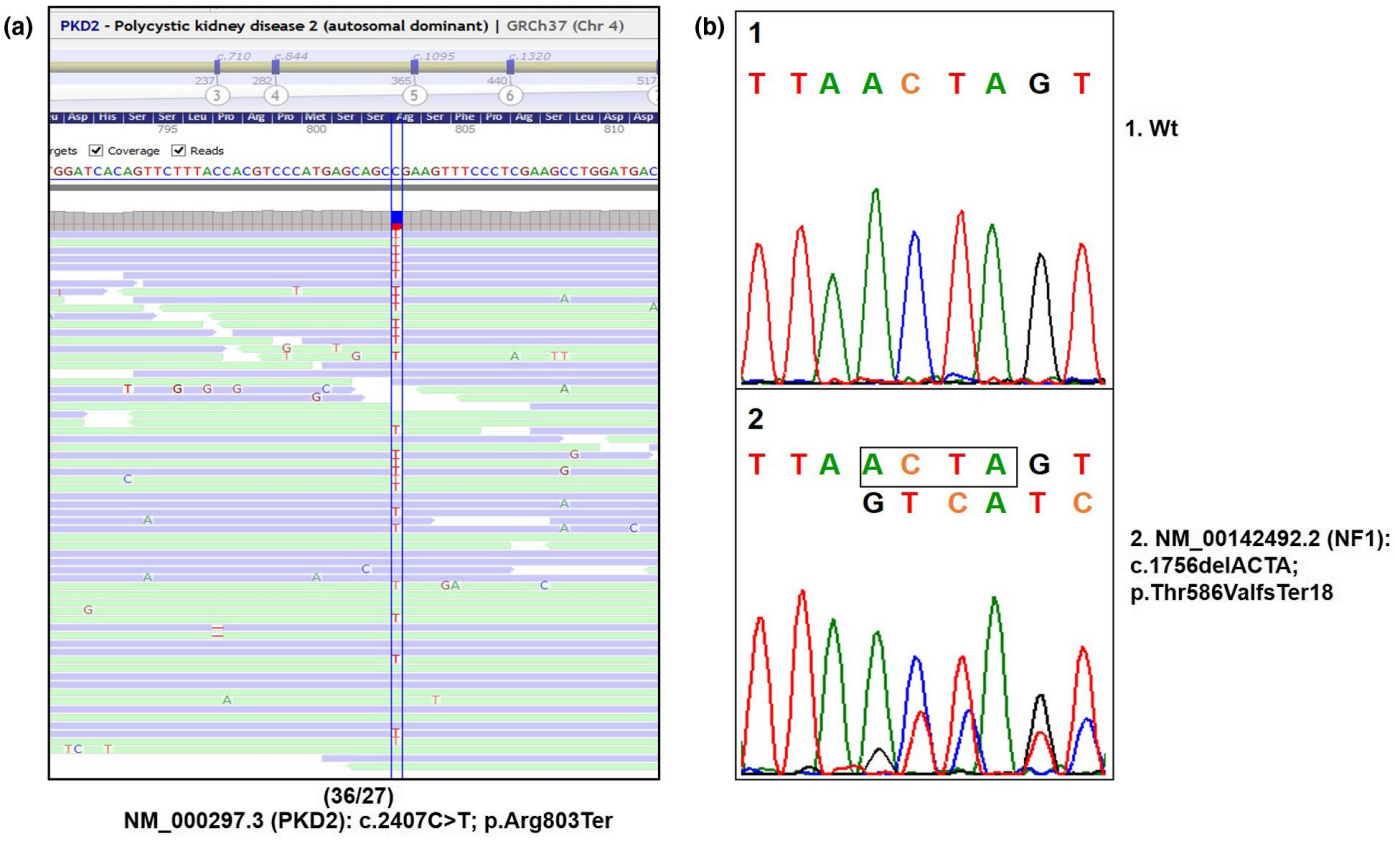
Molecular establishment of both mutations in the proband. (a) NGS PKD2 view of the nonsense mutation at the exon 13; (c.2407C>T; p.Arg803Ter). (b) Sanger sequencing of mutated NF1 allele; (c.1756delACTA; p.Thr586ValfsTer18) at exon 12a, yielding a putative truncated neurofibromin. NF1, neurofibromatosis type 1; NGS, next‐generation sequencing

The pedigree of the family is plotted in Figure 2. Tracing the family history of the proband (III‐2), several members, including his mother (II‐1), maternal grandmother (I‐1), and maternal aunt (II‐3) were all affected by ADPKD. His older brother (III‐1) was found to have also ADPKD at the age of 14 years. Consistent with linkage results, ADPKD affected brother and other affected family members do not share the same *PKD1* maternal haplotype. This result may it initially focused in *PKD2* gene. In fact, from the four microsatellite markers used for *PKD2*, one of them (JV106, next to the gene) was shared with the affected brother, and not with definitively unaffected members of this family (data not shown). The rest of microsatellites resulted non‐informative. This weak haplotype segregation implied later the use of our NGS custom designed targeted panel for inherited nephropathies (Nefroseq v1.1). We detected in our proband a previously described nonsense heterozygous *PKD2* mutation NM_000297.3:c.2407C>T; p.Arg803Ter (Figure 3a) in exon 13, resulted in a truncated polycystin 2 protein, that was definitely pathogenic (Mayo’s Clinic ADPKD mutation database; http://pkdb.mayo.edu/ and LOVD, HGMD, Clin Var) and reported in different ethnicity (Audrezetet al., 2012; Choi et al., 2014; Chung et al., 2006; Deltas, 2001; Zhang et al., 2005). This mutation changed the Arg417 codon, CGA, to a stop codon, TGA, resulting in a predicted truncated protein 803 amino acids shorter than normal (Zhang et al., 2005). ACMG/AMP classification was pathogenic (PVS1, PM2, PP3, PP4, PP5), with several in silico predictors as damaging (DANN, CADD, Mutation Taster, FATHMM‐MKL, LRT, EIGEN, EIGEN PC) and highly conserved (GERP). The variant was not previously observed in European non‐Finnish (genomAD; Exomes and Genomes). This pathogenic variant was validated by Sanger sequencing, as well as other available affected family members (the proband`s mother, proband`s aunt, and proband`s older brother).

### 3.2 NF1 genetic

Moreover, the proband (III‐2) presented café‐au‐lait spots at birth, which increased in number and size in the first years of life associated to axillary and inguinal freckling. He was later found to have optic gliomas, confirming the presence of NF1. There was no family history of NF1 and his two brothers have no stigmata at all of neurofibromatosis, suggesting that a de novo* NF1* mutation occurred in the proband. Mutational analysis of *NF1* gene found a 4‐bp deletion NM_001042492.3:c.1756_1759delACTA; p.Thr586ValfsTer18 at exon 12a, yielding a putative truncated neurofibromin (Figure 3b). The variant has been previously described in different ethnic groups (Ars et al., 2003; Nemethova et al., 2013; Park & Pivnick, 1998; Pemov et al., 2017; Yap et al., 2017). ACMG/AMP classifies this change as pathogenic (PVS1, PM2, PP3, PP4, PP5). Population frequencies is low in European non‐Finnish (1/311502). This mutation was detected neither in his parents nor in his brothers.

Therefore, the coexistence of these two disorders in this male may be simply the coincidental inheritance of the maternal *PKD2* gene mutation (at chromosome 4q22.1) and de novo mutated *NF1* gene in chromosome 17q11.2 band, which seemed to be associated with optic gliomas formation.

### 3.3 ADPKD progression

Our proband (III‐2) showed that his kidney size (htTKV) does not differ significantly from his older brother with ADPKD only (III‐1) and from two ADPKD cohorts: (PKD2 patients) and (ADPKD without hypertension) (Table 2). However, the annual rate of htTKV growth in the proband was approximately 50% when compared with his older brother and with these two ADPKD cohorts. In addition, in both brothers and two control ADPKD cohorts, (PKD2 patients) and (ADPKD without hypertension) the htTKV and the annual rate of htTKV growth were smaller than in other cohort (ADPKD ≤ 40 years). Moreover, in both brothers the eGFR_CysC_ was not different that in our three control ADPKD cohorts. Moreover, both brothers were classified as 1C class (moderate risk of progression) and the prediction of future eGFR based on Mayo imaging classification was superimposable in both (ESRD at 64 and 63 years, respectively).

## 4 DISCUSSION

### 4.1 NF1 and genotype‐phenotype correlations

The principal clinical features of NF1 disease are café‐au‐lait spots, neurofibromas, Lisch nodules (hamartomas) of the iris, axillary/inguinal freckling and optic gliomas. It is characterized by a highly variable expressivity and age‐dependent clinical features, including vascular abnormalities that are also a recognized characteristic of neurofibromatosis (Alkindy, Chuzhanova, Kini, Cooper, & Upadhyaya, 2012; Bargiela et al., 2018; Hitchcock & Gibson, 2017; Kebudiet al., 2008; Kurtelius et al., 2017; Rasmussen, Yang, & Friedman, 2001; Stewart et al., 2018; Sylvester, Drohan, & Sergott, 2006). Thus, NF1 patients under 29 years of age have an increased prevalence of intracranial aneurysms relative to the general population risk (Hitchcock & Gibson, 2017). On the other hand, the incidence of pheochromocytoma in NF1 is estimated between 0.1% and 5.7%, where 22% are asymptomatic. In autopsy series the incidence is higher, between 3% and 13%, and the median age of pheochromocytoma presentation in one previous study was 43 years (Gorgel et al., 2014; Walther, Herring, Enquist, Keiser, & Linehan, 1999). Furthermore, just about half of people with NF1 do not have plexiform neurofibromas, but most are internal and not suspected clinically (Mautner et al., 2008; Plotkin et al., 2012; Tonsgard, Kwak, Short, & Dachman, 1998) and also most of these tumors grow slowly. When symptomatic, plexiform neurofibromas can cause disfigurement and may compromise function affecting quality of life.

The *NF1* gene codes for a protein known as neurofibromin, which is expressed in many tissues, including brain, kidney, spleen, and thymus and is involved in inhibiting cell proliferation. Among its many roles, the most important function can be the tumor suppression through the cellular GTPase systems (Abramowicz & Gos, 2014; Hirbe & Gutmann, 2014; Ward & Gutmann, 2005). In fact, mutations in the *NF1* gene result in loss of functional protein, causing the wide spectrum of clinical findings including NF1‐associatedtumors. Interestingly, only approximately 15%–20% of patients with NF1 develop optic glioma, sometimes as the first sign of subtle NF1 (Kebudiet al., 2008) and 10% of these children will have tumor progression (Sylvester et al., 2006). Approximately one‐half of the cases of NF1 are familial; the remainder are sporadic cases, due to de novo mutations in *NF1* gene (Ward & Gutmann, 2005). A 360–amino acid region of the gene product shows homology to the catalytic domain of the mammalian GTPase‐activating proteins (GAP). This region is referred to as the NF1‐GAP–related domain (NF1 GRD) and is encoded by the central portion of the *NF1* gene, which encompasses exons 21–27a. The GAP proteins downregulate the activity of the RAS oncogene by stimulating its intrinsic GTPase activity that converts the active, guanine triphosphate (GTP)‐bound form of p21 Ras to the inactive guanine diphosphate–bound form in the very early stages of the signal transduction cascade. Among the numerous NF1 mutations reported to date, very few of them correlate with the NF1 tumor phenotype (Alkindy et al., 2012). Functional studies showed that NF1‐GRD mutations reduced the GAP activity from 200‐ to 400‐fold, but no relationship has been demonstrated with the NF1 phenotype (Deltas, 2001). In fact, the frameshift mutation deletion in exon 12a, c.1756_1759delACTA, found in our proband seemed to be associated with the optic glioma observed at early age. As our patient, other patients with optic gliomas had the same mutation; c.1756_1759delACTA (Park & Pivnick, 1998; Rasmussen et al., 2001), which is outside the GRD. In addition, in one of previous studies, all patients with optic glioma harbor mutations yielding truncated neurofibromins (Park & Pivnick, 1998). However, c.1756_1759delACTA mutation does not necessarily correlate with tumor phenotype. Moreover, absence of mutations in the GRD region in other patients who developed tumors indicates that other regions of neurofibromin are probably also involved in modulating *ras* GTPase activity. Indeed, modifying genes may be involved in the variability of NF1 expression. This is supported by the observation that identical NF1 specific mutations do not cause the same phenotypes in unrelated NF1 patients. Furthermore, our patient did not have plexiform neurofibromas, whereas other patients wearing the same change (c.1756_1759delACTA), describe the presence of these (Ars et al., 2003; Park & Pivnick, 1998). In addition, our patient was under 30´s, had normal blood pressure and different image testing did not show any trace of pheochromocytoma (Gorgel et al., 2014; Walther et al., 1999). Finally, to be remarked that regarding these genotype‐phenotype correlation‐ships is that our patient currently, had mild‐moderate phenotype of NF1‐disease with no plexiform neurofibromas, neither pheochromocytomas nor other tumor‐associated complications, and stable and controlled optic gliomas. No specific phenotype can be associated to this variant observed in our subject. At this point only NF1 microdeletion patients present a more severe phenotype than that observed in classical NF1 patients, particularly in respect to the presence of early appearance of cutaneous neurofibromas, more frequent and more severe learning disabilities, somatic overgrowth, and dysmorphic facial features, among others (Koczkowska et al., 2020; Messiaen et al., 2011; Pasmant et al., 2010).

### 4.2 ADPKD progression

The hallmark characteristic of ADPKD is the progressive development and expansion of cysts in the kidney leading to ESRD. It contributes to approximately 10% of ESRD patients in Europe (Cornec‐Le Gall et al., 2017; Willey et al., 2017). In addition, it can be associated with several extrarenal manifestations including hypertension, symptomatic extrarenal cysts, and subarachnoid hemorrhage from intracranial aneurysms (Audrézet et al., 2016; Hitchcock & Gibson, 2017; Magistroni et al., 2003; Rossetti et al., 2002). The vast majority of the patients develop the disease between the ages of 20–40 years, but, notably, among a small proportion (2%–5%) of ADPKD patient symptoms can manifest during childhood or even prenatally (“early onset,” before 15 years old) or even in utero (“very early onset”) (Audrézet et al., 2016). Moreover, there is evidence of genotype‐phenotype correlation in ADPKD. Mutations in *PKD1* cause more severe disease than *PKD2*, with ESRD occurring approximately 20 years earlier, at an average age of 58 years versus. 79 years. In addition, carriers of truncating mutations in *PKD*1 reach ESRD approximately 12 years prior to those with missense mutations (Harris & Rossetti, 2010). Therefore, disease progression of ADPKD is highly variable, in part because of a strong gene locus effect (Rossetti et al., 2002; Magistroni et al., 2003; Audrézet et al., 2016; Harris & Rossetti, 2010) or by a modifier effect, as it has been suggested by a significant intrafamilial renal disease variability in ADPKD (Cornec‐Le Gall et al., 2017; Cornec‐Le Gall et al., 2018).

### 4.3 Co‐occurrence of ADPKD and NF1

**FIGURE 4:**
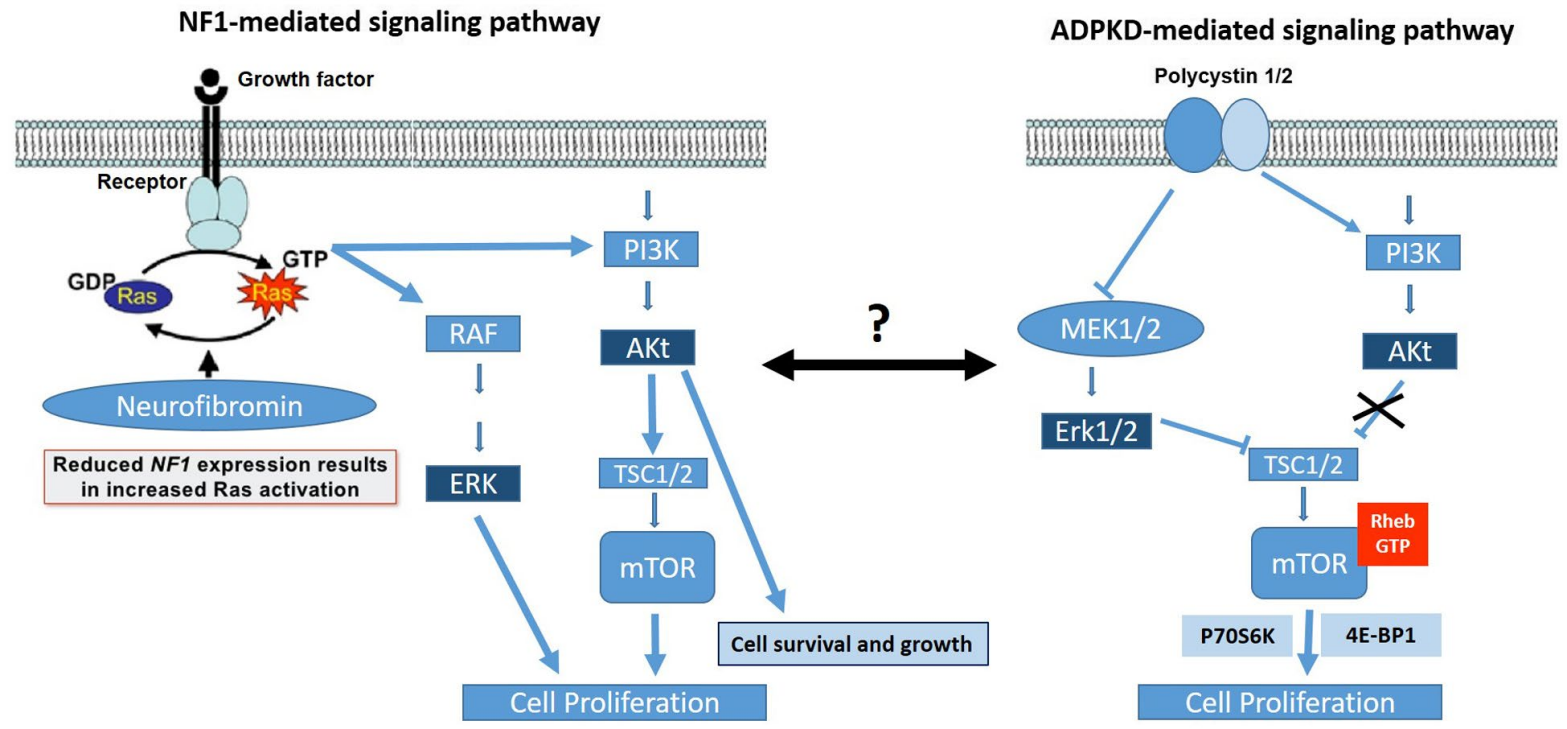
Schematic view of cell growth and proliferation molecular pathways involved in neurofibromatosis type1 (NF1) (left) and autosomal dominant polycystic disease (ADPKD) (right) by GTPase signaling

A single individual carrying mutations in two different genes causing two different diseases, and, therefore, suffering both entities, is not common but depends on the prevalence of each disease. Because the prevalence of ADPKD in the general population is 1 in 500–1,000 (Torres et al., 2007) (whereas PKD2 genetic prevalence is estimated at 2.31/10,000) (Choi et al., 2014) and the prevalence of NF1 is approximately 1 in 3,000 (Ward & Gutmann, 2005), concurrence of both diseases (ADPKD and NF1) is predicted to occur in 1 in 1,500,000–3,000,000 individuals; concurrence of PKD2 disease and NF1 disease is predicted to occur in 1/13,043,400. Therefore, occurrence of both, ADPKD and NF1, in a single individual is very rare. In fact, only six patients have been reported in the literature (Chen et al., 2002; Flego et al., 2003; Siegelman et al., 1971; Varma et al., 1982; Table 1). Chen et al. (2002) thought that the co‐occurrence of these two disorders together, in three members of a family, was due a de novo mutation in *NF1* gene that occurred in the ADPKD mother who transmitted both diseases to her two sons. Flego et al. (2003) reported a patient who inherited NF1 and ADPKD from his mother and father, respectively. However, in just one of these cases was assumed a sporadic mutation for *NF1* gene and the inheritance of other condition, and in only two cases this association was related with tumor formation, but none of them with optic gliomas. Therefore, all of these reports, as in our case, it may be an association by chance. However, although ADPKD and NF1 are due to alterations in two different and independent genes, we cannot rule out the possibility of a genetic or biochemical interaction and/or causal relationship between these two anomalies. In both, ADPKD and in NF1 disease loss of heterozygosity are involved as the mechanism responsible for disease expression. Although *PKD2* and *NF1* genes are not apparently located closely at the genome, their functions at the cellular level are intimately related (see Figure 4). They both are involved in inhibiting cell proliferation and differentiation through the GTPase systems (Abramowicz & Gos, 2014; Delmas et al., 2002; Hirbe & Gutmann, 2014; Kim et al., 1999). In view of the similarity of the underlying biochemical abnormality in PKD2 and NF1, the presence of both conditions in a single individual might lead (under this hypothesis) to a more severe disease particularly in terms of tumor progression. Therefore, this might have resulted in additive or synergistic effect of dysfunctional tumor‐suppressor genes in rapid progression of optic gliomas in this unusual patient (Abramowicz & Gos, 2014; Erbay et al., 2004; Hirbe & Gutmann, 2014). In addition, in both diseases has been reported an associated high risk of intracranial aneurysms (ADPKD, 4%–17%; NF1, 9%–11%) (Erbay et al., 2004) and in case of associated mutations of the neurofibromin and polycystin‐2 proteins it is expected to have additive or synergistic intracranial aneurysms disease presentation and/or associated tumors progression. However, inferred by our clinic observations, he did not appear to be more severely affected than is typically reported with either condition alone and in fact, we did not detect any intracranial aneurysms. Thus, the absence of a synergistic effect may be explained by the underlying molecular function of the neurofibromin and polycystin‐2 proteins. Indeed, loss of neurofibromin function reduces control of Ras and aberrant activation of the mTOR pathway leading to increased cell growth (Johannessen et al., 2005, 2008). Loss of polycystin‐2 function leads to increased cAMP activity, and hyperactivation of mTOR and S6K (Delmas et al., 2002; Gogusev et al., 2003; Kim et al., 1999). While neurofibromin and polycystin‐2 act to restrain cell growth, they appear to be acting in a separate way. Thus, unlike astrocytes, in which cAMP acts as an antimitogenic signal, cAMP is a mitogen for Schwann cells, and neurofibromin functions to antagonize, rather than stimulate, cAMP production (Figure 4). Therefore, as it is denoted in our patient individuals with both *PKD2* and *NF1* mutations seemed not have a greater risk for tumor and/or cerebral aneurysm formation than expected with either condition alone. However, more individuals will need to support this fact. Moreover, the evidence that mTOR deregulation participates in NF1‐related tumorigenesis and the recent finding that mTOR and its downstream effector S6K play a critical role in the pathogenesis of ADPKD, support the possibility that rapamycin could represent a viable therapy for patients with this rare association (Hegedus et al., 2008; Johannessen et al., 2005, 2008; Shillingford et al., 2006; Wahl et al., 2006; Wen & Kesari, 2008).

The case we present here has a de novo deletion in exon 12a of the *NF1* gene and an inherited nonsense mutation in exon 13 of the *PKD2* gene. Although the functional consequences of *NF1* mutation on ADPKD is not known, the absence of great differences on renal volume and renal function in the proband with respect to his older brother with ADPKD only and two ADPKD cohorts, is strongly suggestive of that *NF1* mutation do not have a pathogenic additive effect on ADPKD. In contrast, the difference in annual rate of htTKV growth between the proband and his older brother with ADPKD only and with two ADPKD cohorts may suggest a protective effect. Moreover, both brothers were classified as 1C class (moderate risk of progression) and the prediction of future eGFR based on Mayo imaging classification was superimposable in both (ESRD at 64 and 63 years, respectively), which may be considered atypical for PKD2 (Cornec‐Le Gall et al., 2017; Cornec‐Le Gall et al., 2018). Interestingly, ADPKD in this family was originated by a truncating mutation in *PKD2* and is a fact that the proband`s mother, proband`s aunt and proband`s grandmother presented ESRD at age of 49, 60, and 70, respectively, which may be considered as intra‐familial renal disease variability for PKD2 and may suggest the presence of a modifier effect for rapid progression. So, ADPKD in this family was quite severe for *PKD2* variant (Robinson et al., 2012), but what is most surprising is the apparent limited impact of an additional *NF1* mutation in the proband.

## 5 CONCLUSION AND OUTLOOK

In summary, this is the first reported case of concurrence of a truncated *NF1* mutation with optic pathway gliomas and a nonsense *PKD2* mutation. Despite the presence of both conditions in a single individual, there was not additive or synergistic effect of dysfunctional tumor‐suppressor genes in rapid formation of optic gliomas or in ADPKD progression in this unusual patient. In addition, the widely used of genomic approaches, such as NGS, may allowed us to find co‐concurrence of more than one pathogenic variant in the patients, such as in our proband, which may mask an expected phenotype. Thus, we would like to alert to our colleagues the necessity of checking for more than one pathogenic variant in atypical phenotypes for known diseases.

## CONFLICTS OF INTEREST

The authors declare that there is no conflict of interest regarding the publication of this paper.

## DISCLOSURE

Dr. Peces was an investigator for an Oksuka‐sponsered study investigating tolvaptan in the treatment of ADPKD (REPRISE). The other authors declare that they have not relevant financial interests.

## AUTHORS’ CONTRIBUTIONS

All authors were involved in drafting the manuscript, gave final approval for the version to be published and agreed to be accountable for all aspects of the work in ensuring that questions related to the accuracy or integrity of any part of the work are appropriately investigated and resolved. RP made substantial contributions to the concept/design and acquisition, analysis, and interpretation of data. RM, YM, CH, CP, DT, EC, RS, PL, and JN made substantial contributions to the acquisition, analysis, and interpretation of data.

## ETHICS APPROVAL AND CONSENT TO PARTICIPATE

This article was conducted in accordance with the World Medical Association Declaration of Helsinki, all its amendments and national regulations.

## CONSENT FOR PUBLICATION

Written informed consents were obtained from the patients for publication of this article.

## Data Availability

The dataset supporting the conclusions of this article are included within the article.
